# A Review on Microbial Species for Forensic Body Fluid Identification in Healthy and Diseased Humans

**DOI:** 10.1007/s00284-023-03413-x

**Published:** 2023-07-25

**Authors:** Mishka Dass, Yashna Singh, Meenu Ghai

**Affiliations:** Department of Genetics, School of Life Sciences, University of KwaZulu Natal, Westville Campus, Private Bag X 54001, Durban, KwaZulu-Natal South Africa

## Abstract

Microbial communities present in body fluids can assist in distinguishing between types of body fluids. Metagenomic studies have reported bacterial genera which are core to specific body fluids and are greatly influenced by geographical location and ethnicity. Bacteria in body fluids could also be due to bacterial infection; hence, it would be worthwhile taking into consideration bacterial species associated with diseases. The present review reports bacterial species characteristic of diseased and healthy body fluids across geographical locations, and bacteria described in forensic studies, with the aim of collating a set of bacteria to serve as the core species-specific markers for forensic body fluid identification. The most widely reported saliva-specific bacterial species are *Streptococcus salivarius*, *Prevotella melaninogenica*, *Neisseria flavescens,* with *Fusobacterium nucleatum* associated with increased diseased state. *Lactobacillus crispatus* and *Lactobacillus iners* are frequently dominant in the vaginal microbiome of healthy women. *Atopobium vaginae, Prevotella bivia,* and *Gardnerella vaginalis* are more prevalent in women with bacterial vaginosis. Semen and urine-specific bacteria at species level have not been reported, and menstrual blood bacteria are indistinguishable from vaginal fluid. Targeting more than one bacterial species is recommended for accurate body fluid identification. Although metagenomic sequencing provides information of a broad microbial profile, the specific bacterial species could be used to design biosensors for rapid body fluid identification. Validation of microbial typing methods and its application in identifying body fluids in a mixed sample would allow regular use of microbial profiling in a forensic workflow.

**Supplementary Information:**

The online version contains supplementary material available at 10.1007/s00284-023-03413-x.

## Introduction

One of the emerging applications of microbial profiling in forensic sciences is its use in distinguishing between body fluids. Identification of body fluids such as saliva, semen, urine, and vaginal fluid is important for the reconstruction of crime scenes [1, 2]. For example, detection of saliva on blood stains allows distinguishing of expectorated blood spatter [3]. The identification of semen and vaginal fluid mixture indicates sexual assault crimes [4, 5]. Identification of urine at crime scenes suggests an incontinent victim or suspect. Compared to other body fluids, urine is the least viscous and is likely to absorb quicker. It cannot be visibly seen in a mixed sample, and therefore, microbial profiling could be used for identification [6].

Microbial profiling generally focuses on housekeeping genes such as the 16S rRNA gene. The 16S-23S rRNA intergenic spacer is a noncoding region that is also targeted to design markers to identify bacterial species [7]. In addition, other genes targeted for species-specific identification include the glucosyltransferase (*gft*) gene and the chaperonin-60 (*cpn60*) gene [8, 9].

Till date, articles published on body fluid identification using microbial profiling have focused on identifying the following body fluids: saliva, vaginal fluid, semen, peripheral blood, menstrual blood, fecal matter, as well as samples from skin and nasal secretions [2, 10, 11]. The present review aimed to compare available literature on the microbial profile of saliva, vaginal fluid, menstrual blood, semen, and urine across geographical locations in forensic and disease studies, and to collate a set of bacterial species specific to body fluids in healthy and diseased humans.

### Forensic Body Fluid Identification

There are currently several methods used to identify body fluids, such as chemical tests, enzymatic assays (protein catalytic activity tests), immunological tests, spectroscopic methods, mRNA tests, microscopy, and microbial profiling [4–6, 12–14]. Body fluid identification tests fall into one of two categories: presumptive tests and confirmatory tests. Presumptive testing is done for preliminary identification of the body fluid, and confirmatory testing is used to confirm the identity of the body fluid. Often, the catalytic, enzymatic, and immunological testing methods have limitations such as sample destruction, low sensitivity, and high rates of false positive and negative results. Enzymatic methods can be affected by enzyme degradation when exposed to heat, chemicals, mold, and the breakdown of organic material [5, 6].

DNA methylation markers are specific and sensitive, as they target the tissue-specific DNA methylation patterns for the identification of body fluids [15, 16]. However, methylation patterns can be affected by age, diet, and lifestyle choices, such as smoking [15].mRNA profiling also allows specific identification of relevant body fluids such as semen, blood, saliva, menstrual blood, and cervicovaginal fluid. RNA methods allow coextraction of both RNA and DNA from one sample [17]. One major disadvantage of mRNA profiling is that mRNA is sensitive to degradation when exposed to harsh environmental conditions such as humidity and UV light. MicroRNAs (miRNAs) have been demonstrated to be less susceptible to degradation than mRNA in most cases [18]. Microbial profiling could offer a suitable complement to the existing molecular identification methods because bacterial DNA is less sensitive to degradation by environmental factors as compared to human DNA and can persist longer on surfaces [19, 20].

The Human microbiome project has reported distinct microbiome signature in different body fluids, especially in saliva, vaginal fluid, and fecal matter, with the microbiome signature associated with each body fluid being distinct, stable, and predictable [21]. Body fluid identification by microbial profiling is still in its infancy and is not yet a common practice in forensic science laboratories because there are currently no standards that exist for microbial profiling for forensic purposes [7]. The development of the standards by accredited organizations would allow microbial profiling to carry more weight in criminal cases [7, 22].

#### Disadvantages of Microbial Profiling (change font size to match other headings)

Different areas of the human body harbor different microbial species, which can change during the course of a human’s life [23]. For example, the microbial species found in the vaginal region can vary throughout the menstrual cycle [4, 12]. Vaginal and urinary tract infections, which are common in females, can alter the type of bacterial species found in vaginal fluid and urine at the time of infection [4]. A reduction in *Lactobacillus* taxa has been specifically observed during post menopause. [24]. Geographical location has a crucial effect on salivary microbiota, more than age, gender, or smoking, although smoking status has a significant effect on the microbiome [25].

## Forensic Microbiome Database

The forensic microbiome database (FMD) is composed of publicly available 16S rRNA-sequencing data and metadata from various human body sites. Users can access the website to analyze the taxonomic differences between microbiomes from different locations and predict geolocations of their own data by using existing FMD sequences [26].

FMD lacks global representation as only a few countries in Africa and the Middle East are covered for data collection [26]. FMD currently has data obtained from USA, Australia, Italy, South Africa, and United Kingdom for the microbiome of the vagina. Microbiome data from saliva are available for India, Italy, Japan, South Korea, and USA. The USA is the only country to have data collected on urine [27]. While FMD has several well-distinguished categories of body sites to analyze, there are no data available for seminal fluid, which is a key body fluid in forensic criminal investigations.

Most of the existing studies conducted either for forensic purposes, understanding the human microbiome of a specific body site, or healthcare studies such as microbial changes responsible for diseases, have targeted body fluids collected from USA, Europe, Iran, Japan, Turkey, and China.

The section below discusses studies on the microbial profile of saliva, urine, semen and vaginal fluid across different geographical locations and in disease and forensic settings.

### Bacteria for Identification of Saliva, Vaginal Fluid, Semen, Menstrual, Blood and Urine

A core microbiome is a set of bacteria which defines a specific body fluid or site, regardless of disease state and environmental factors [28]. Salivary core microbiomes have been found to be greatly influenced by geographical location and shared environment [25], which could be due to differences in dietary patterns [29].

#### Saliva

Recent studies have identified *Streptococcus*, *Neisseria*, and *Prevotella* as the dominant genus of the healthy core human salivary microbiome [28, 29]. The common saliva-specific species include *Streptococcus salivarius, Streptococcus oralis* [8] *Veillonella atypica*, *Prevotella melaninogenica*, *Prevotella nigrescens*, *Neisseria meningtidis*, *Haemophilus influenza, Fusobacterium nucleatum,* and *Lactobacillus salivarius* [22, 30, 31] (Table S1). A 16S rRNA pyrosequencing study in a cohort of 27 monozygotic and 18 dizygotic twin pairs consisting of Non-Hispanic Whites (93.5%), Hispanic Whites (1.9%), Hispanics of unknown race (3.7%), American Indians (0.9%), and multi-ethnics (0.9%) showed that eight bacterial genera (*Streptococcus, Veillonella, Gemella, Granulicatella, Neisseria*, *Prevotella, Rothia, and Fusobacterium*) represent the core saliva microbiome, and were observed in >95% of samples [25] (Table S1). In a larger study on 900 Finnish children, aged 11–14 years, the saliva microbiota composition and abundance were significantly associated with body size and gender. The core salivary microbiota consisted of genus *Veillonella, Prevotella, Streptococcus, Selenomonas, and Neisseria.* The core bacteria decreased in overweight and obese children [32].

Ion PGM sequencing of 2343 Japanese adults, aged ≥40 years revealed the following species *Streptococcus mitis*, *Streptococcus salivarius*, *Granulicatella adiacens*, *Neisseria flavescens*, *Rothia mucilaginosa,* and *Prevotella melaninogenica* as saliva specific. Both healthy and diseased individuals (with oral diseases) were included in the study, hence, the study depicted common core bacterial species shared between healthy and diseased Japanese population [33]. In a Chinese cohort, from different geographical regions in China, the following genera were dominant in saliva: *Streptococcus*, *Rothia* and *Neisseria, Granulicatella,* and *Porphyromonas* [34].

##### Forensic Studies

Application of microbial profiling for forensic identification of body fluids requires specificity of the bacterial species to a single body fluid, and sensitivity of its detection in a mixture of body fluids. [35] compared the microbiome profile of the saliva transmitted on a victim’s breast skin and saliva samples from the male suspects, within the first 48 h after a sexual assault. It was found that among male saliva samples, bacteria genera *Fusobacterium*, *Streptococcus*, *Neisseria*, *Haemophilus*, *Porphyromonas*, *Rothia*, *Prevotella,* and *Veillonella* constituted 86.15% of the total bacterial population, whereas in saliva mixed with a victim’s breast skin, the eight bacterial genera constituted 76.72% of the total bacterial population. [35] (Table S1). The study highlighted that the bacterial DNA in saliva can be recovered from saliva transmitted on breast skin within at least 48 h and can link the victim to the crime.

*Streptococcus salivarius* was detected in all the tested samples in a study which targeted the glucosyltransferase gene (*gtf*) to identify the presence of saliva in forensic samples. *Streptococcus salivarius* was not detected in vaginal fluid, semen and urine [11]. Similarly, *Streptococcus salivarius* was found in 90% of the saliva samples and was not detected in blood, semen, vaginal fluid, and menstrual blood [15]. In addition, *Streptococcus salivarius* was the most suitable and robust marker for the identification of aged and forensically exposed saliva stains when compared to other oral bacterial markers [20].

Saliva samples from 140 Korean individuals showed presence of *Streptococcus salivarius*, *Streptococcus sanguinis* and *Neisseria subflava* species in 91.4% of the samples [3]. At least two bacterial species could be detected in all saliva samples. All three bacterial species were identified in 82.5% of 40 mock samples consisting of cigarettes, mugs, straws, paper cups, forks, bite marks, and corncobs. Samples were considered positive for saliva if two or more bacterial species were present.

However, in a study by [12], *Streptococcus salivarius* was also detected in fecal matter (forensic sample), vaginal fluid (forensic sample), and in 78% of pure saliva samples, but was not present in pure vaginal fluid and yoghurt samples.

##### Oral Diseases

The human oral cavity is composed of numerous microbiotas maintaining a balance within the oral cavity. A disruption of this balance often results in an onset of infections caused by an increase in certain bacterial species. Dental caries and periodontitis are the two most commonly occurring diseases in the oral cavity of humans. Dental caries is caused by tooth-adherent harmful bacteria, while periodontitis is a bacterial infection of the mouth and gums which causes the tissue of the mouth to weaken, especially the gums, leading to tooth loss. According to the World Health Organization (WHO), an estimated 2 billion people were infected with dental caries in 2022, while the global population affected by periodontitis is estimated to be between 10 and 15% [36, 37].

*Streptococcus mutans is* found mostly in individuals with dental caries [8, 38]. *Lactobacillus salivarius* is mostly associated with saliva of healthy individuals [30]; however, in a study by [39], *Lactobacillus fermentum*, *Lactobacillus salivarius,* and *Lactobacillus rhamnosus* were the most dominant bacterial species identified from the saliva of patients with dental caries.

*Fusobacterium nucleatum* is predominantly present in the saliva of individuals suffering with periodontitis [40] but is also found in healthy individuals. Additional studies using Illumina MiSeq 16S rRNA amplicon sequencing confirmed that *Fusobacterium* increases with the progression of periodontal disease [41].

The saliva of 134 sexually active participants from Puerto Rico, aged 21 to 49 years old, who had varying degrees of periodontal diseases was analyzed. Illumina MiSeq amplification sequencing revealed that *Prevotella*, *Veillonella*, and *Porphyromonas* increased when disease severity was higher, while *Neisseria* decreased [41].

A study was conducted to evaluate the changes in the saliva microbiome profile of individuals with severe periodontitis after treatment had been administered. Results showed that the broader microbiome profile of saliva did not change after the treatment but could show variation at the species level. *Streptococcus salivarius* and *Streptococcus mitis* were the most dominant species in saliva. The most prevalent bacterial species associated with periodontitis were *Prevotella melaninogenica* and *Porphyromonas pasteri* [36].

Colorectal cancer is the third most frequent malignancy in the world with an estimated 1.9 million global cases in 2020 [42]. Colorectal cancer patients have increased amounts of *Fusobacterium nucleatum* present in their saliva when compared to healthy controls (*p* = 0.001) [43] (Table 2). Similar findings were reported by [41, 44].

**Fig. 1:**
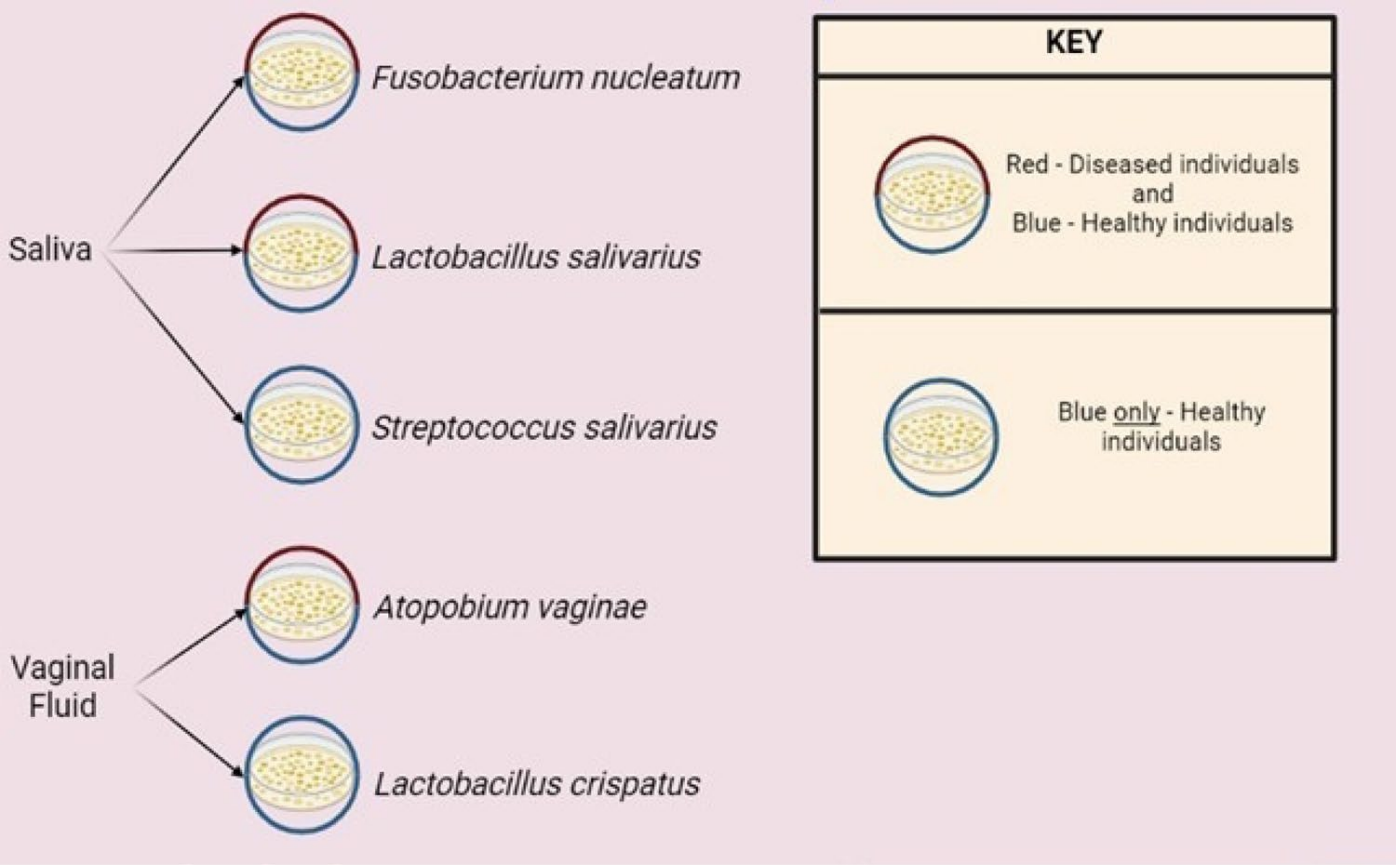
Set of bacterial species which could be targeted for identification of saliva and vaginal fluid, blue indicates bacterial species found in healthy individuals, and red and blue indicates bacterial species found in both healthy individuals and diseased individuals. (Created using Biorender.com)

Based on the above knowledge, the following species are specific to saliva: *Streptococcus salivarius*, *Prevotella melaninogenica*, *Neisseria flavescens, and Fusobacterium nucleatum*, as they are found frequently among healthy and diseased individuals with *Fusobacterium nucletum* occurring more frequently in the diseased state (Table 1, Fig. 1).

**Table 1:** Bacterial species frequently found in saliva and vaginal fluid based on the studies described in the text

No	Body fluids	Bacterial species
1	Saliva	*Fusobacterium nucleatum* [39, 40, 43, 44]
2	Saliva	*Streptococcus salivarius* [8, 20, 36]
3	Saliva	*Prevotella melaninogenica* [31, 33]
4	Saliva	*Neisseria flavescens* [33]
5	Vaginal fluid	*Atopobium vaginae* [4, 45–48]
6	Vaginal fluid	*Gardnerella vaginalis* [45–48]
7	Vaginal fluid	*Lactobacillus crispatus* [4, 12, 46–50]
8	Vaginal fluid	*Lactobacillus iners* [46, 51, 48]

#### Vaginal Fluid

The vaginal microbiome in healthy women of reproductive age is dominated by the *Lactobacillus* genus. *Lactobacilli* are essential in maintaining a healthy vaginal equilibrium by preventing the colonization of disease-causing microbiota. Majority of vaginal microbiomes are composed mainly of either one of the following *Lactobacillus sp*.: *Lactobacillus crispatus, Lactobacillus jensenii, Lactobacillus iners, or Lactobacillus gasseri.* [45].

The vaginal microbiome can be influenced by ethnicity, pregnancy, menstrual cycle, exercise, diet, hormones, an individual’s immune system, the use of contraceptives, use of antibiotics, sexual activities, genetic differences, and disease [46, 52, 53].

A study was conducted to gain a better understanding of the vaginal microbiome by using 1312 publicly available datasets from metagenomic sequencing studies of healthy vaginotypes and their microbial composition. The dominant genus among all samples was *Lactobacillus* with an average abundance of (68.35% ± 38.09%), followed by *Gardnerella* (7.42% ± 17.53%), *Vibrio* and *Atopobium* (2.99% ± 14.43%). At the species level, *Lactobacillus crispatus* was the most abundant (41.52% ± 42.63%). *Lactobacillus* species have been reported to immediately restore colonization after vaginal microenvironment damage [47].

A comparative metagenomic study was done on the vaginal microbiome of healthy women to analyze taxonomy, functional levels and microbial communities' genome content. Eighty-two in-house sequenced datasets from Chinese women were compared with 133 randomly selected Amercian metagenome datasets in the Human Microbiome Project (HMP1-II) cohort [46]; 111 species were identified, and the most dominant genera present were *Lactobacillus* including *Lactobacillus crispatus*, *Lactobacillus iners*, *Lactobacillus gasseri, Lactobacillus jenseii,* and *Atopobium vaginae, Gardnerella vaginalis* and *Prevotella amnii *(Table S1)*.* Results from this study were compared to 133 different ethnicity datasets from the human microbiome project, where results showed differences between Chinese and American women, with *Atopobium vaginae* and *Prevotella amnii* more prevalent among Chinese women. *Lactobacillus crispatus* and *Lactobacillus iners* were significant contributors to the variation in community abundance (*p* < 0.001;* R*^2^ > 0.98).

The vaginal microbial community of healthy women at pre-pregnancy and during pregnancy is dominated by *Lactobacillus crispatus*, but at the puerperium (6 week period after birth), decreased abundance of protective *Lactobacillus* species was observed, which makes one vulnerable to diseases. Additionally, vaginal pH was reported to be an important factor affecting the vaginal microbial community [54].

##### Forensic Studies

On the forensic front, *Lactobacillus crispatus* was detected in 52% of pure vaginal fluid samples and in 83% of forensic samples (samples collected from female genital regions, cytological microscopic slides, female underwear stored for a lengthy period, and swabs from living and dead subjects). *Lactobacillus crispatus* has been found mainly in vaginal fluid but has also been detected in saliva, (possibly due to a mixture of body fluids collected from crime scenes) [12, 15] and in female urine and menstrual blood, but not in semen [10, 15]. *Lactobacillus crispatus* and *Lactobacillus gasseri* could potentially be used as markers for the identification of vaginal fluid, since both markers were detected in vaginal secretions and were not detected in blood, semen, and saliva [49]. *Lactobacillus iners* was found specific to vaginal fluid in a study from women in China [51].

##### Disease Studies

Globally, 23–29% of women suffer from bacterial vaginosis which is a common disease affecting the lower genital tract [55]. It is caused by a drastic decrease in *Lactobacillus sp*., and an increase of other bacterial species, such as *Atopobium* and *Gardnerella. Gardnerella vaginalis, Atopobium vaginae,* and *Prevotella biva* are found in increased abundance in women with bacterial vaginosis [56–58] (Table 2). *Megasphaera type *2, BVAB1 and *Gardnerella vaginalis* have significantly higher concentrations in bacterial vaginosis samples (*p* < 0.005) in addition to *Atopobium vaginae*. [45].

**Table 2:** List of bacterial species found in body fluids of diseased individuals

Bacterial species	Body fluid	Disease
*Escherichia coli* [59, 60]	Urine	Individuals with UTIs
*Pseudomonas aeruginosa* [59, 61, 62]	Urine	Individuals with UTIs
*Gardnerella vaginalis* [45, 56–58]	Vaginal fluid	Individuals with bacterial vaginosis
*Atopobium vaginae *[45, 56–58]	Vaginal fluid	Individuals with bacterial vaginosis
*Prevotella bivia* [56–58]	Vaginal fluid	Individuals with bacterial vaginosis
*Corynebacterium seminale* [63–65]	Semen	Increased abundance in men with prostatitis
*Streptococcus mutans* [8, 38]	Saliva	Individuals with dental caries
*Fusobacterium nucleatum* [40]	Saliva	Individuals with periodontitis and increased abundance in colorectal cancer patients

*Lactobacillus coleohominis* is another bacterial species present in both healthy individuals and individuals with bacterial vaginosis, although more commonly occurring in women with bacterial vaginosis [66]. In a study comparing the vaginal *Lactobacillus* species among women from the US and India, with and without bacterial vaginosis, it was found that the presence of *Lactobacillus coleohominis* had a significant association with bacterial vaginosis (*p* < 0.0001) [66].

In a study on 426 African women from Kenya, South Africa, and Rwanda, the participants were divided into the following six groups: Reference group, HIV-positive, practicing intravaginal practices, occupation as sex workers, pregnant, and adolescents. qPCR was used to identify vaginal bacterial species and to look at the correlation between vaginal health and bacterial species. Rwanda sex workers had the highest presence of *Gardnerella vaginalis* *p* = 0.006 and the lowest presence of *Lactobacillus jensenii* compared to the other countries *p* = 0.031. In the HIV-positive group, the Lactobacillus genus was present in 80% of women consisting of *Lactobacillus iners* (63%), *Lactobacillus vaginalis* (30%), *Lactobacillus crispatus* (17%), *Lactobacillus jensenii* and *Lactobacillus gasseri* (10%), *Gardnerella vaginalis* and *Prevotella biva* (70%), and *Atopobium vaginae* (47%) [48].

To investigate the vaginal microbiome and to determine whether microbial communities placed an increased risk on HIV and genital inflammation, a study was conducted on 168 women located in two different South African provinces. All women were HIV negative while bacterial vaginosis status ranged from positive, intermediate, or negative. Results showed that *Gardnerella vaginalis* and *Atopobium vaginae* were among the bacterial species associated with inflammation and bacterial vaginosis. There was a significant difference between prevalence of bacterial vaginosis and geographical location (*p* = 0.04) where rates of bacterial vaginosis were higher in Cape Town (51%), while only 28% of women in Johannesburg were affected. *Lactobacillus crispatus* was among the *Lactobacillus* species found to decrease in abundance in women with high cases of inflammation [50].

Based on the above-mentioned studies, the following microbial species are specific to vaginal fluid: *Lactobacillus crispatus* and *Lactobacillus iners*, with both bacterial species being frequently dominant in the vaginal microbiome of healthy women. *Atopobium vaginae, Prevotella bivia,* and *Gardnerella vaginalis* are more prevalent in women with bacterial vaginosis (Table 1, Fig. 1).

#### Menstrual Blood

Menstrual blood contains blood and vaginal fluid. There is an overlap between the bacteria found in menstrual blood and vaginal fluid, hence, the two body fluids cannot be distinguished using bacterial markers [11]. The stability of the vaginal microbiome of healthy Canadian women (*n* = 27) throughout a menstrual cycle, was analyzed using *cpn60*-based microbiota analysis. Vaginal swabs from naturally cycling reproductive-age women were collected weekly through a single menstrual cycle. It was demonstrated that, in healthy women, vaginal microbiomes remained stable throughout their menstrual cycle with abundance of *Lactobacillus crispatus*, *Lactobacillus iners*, and *Lactobacillus jensenii* [9]. *Lactobacillus gasseri* can also be used to identify menstrual blood [15].

On the contrary [67, 68] described that, during menstruation, the normal dominant species of the vagina, *Lactobacillus jensenii* and *Lactobacillus crispatus* decrease while *Gardnerella vaginalis* and *Lactobacillus iners* bacterial species increase and colonize the vaginal environment. When menstruation is over, the normal level of bacterial species is restored in the vagina. *Gardnerella vaginalis* and *Lactobacillus iners* have also been reported in semen samples [4].

Mycoplasma and Ureaplasma are also part of the vaginal microbiota of many clinically healthy women. *Mycoplasma hominis*, *Mycoplasma genitalium*, *Ureaplasma parvum*, and *Ureaplasma urealyticum* are regularly detected in vaginal samples by using culture or taxon-specific PCR methods. However, these species are rarely reported by 16S rRNA gene-based microbiota analysis, most likely because of universal primer bias [69].

In a healthy female Caucasian cohort, metagenomic analysis was done to compare microbial profile of women, using three different contraceptive regimens: non-hormonal methods (*n* = 54), combined oral contraceptive (COC, *n* = 52), or levonorgestrel intrauterine system (LNG-IUS, *n* = 54). Samples were collected during the menstrual cycle to establish the influence of menstrual bleeding, suppressed ovulation and changes in sex hormones on the composition of the microbiome. The dominant species in vagina were *Gardnerella vaginalis* or *Prevotella* during menstruation, which shifted towards a *Lactobacillus* dominated composition throughout the cycle. The type of hormonal contraception did not significantly affect the microbiome composition in the vagina [70].

#### Semen

Semen microbiome is mainly made up of the following genera*: Pseudomonas, Prevotella, Gardnenella, Corynebacterium, Staphylococcus, Streptococcus, Lactobacillus, Veillonella, and Finegoldia* [5] and Proteobacteria (*Haemophilus*, *Burkholderia*) phyla [5] (Table S1). However, the microbiota of semen is still not well understood and requires further investigation [71].

##### Forensic Studies

Illumina MiSeq was used to evaluate the microbiome of semen after indoor environmental exposure. *Staphylococcus* sp. was the most common bacterial species found in seminal fluid. *Corynebacterium seminale* strain IBS B12915 (CIP 104297), *Corynebacterium singulare* DSM 44357, *Corynebacterium minutissimum*, and *Dermabacter hominis* were new bacterial species identified in semen. While the definite microbial community of semen remains unclear, semen samples were well distinguished from vaginal fluid and saliva samples. The study also concluded that the identification of semen requires the use of more than one microbial marker [5].

##### Disease Studies

Many existing studies focus on bacteria associated with causing infertility or disease in men. A study was conducted to investigate the microbial composition of semen and its influence on sperm parameters. The study [71] included 26 samples from healthy individuals and 64 samples from men with at least one abnormality related to spermatozoa concentration, spermatozoa count, spermatozoa motility, spermatozoa morphology and progressive spermatozoa motility. *Prevotella’*s relative abundance was increased in samples with defective sperm motility while *Staphylococcus* was increased in the corresponding control group. An increased relative abundance of *Lactobacillus* was observed in samples with normal sperm morphology. No difference in microbial richness or diversity was observed between healthy and infertile men [71] (Table S1).

Prostatitis is an infection of the prostate gland, causing inflammation and pelvic pain. Several studies have identified *Corynebacterium seminale,* also known as *Corynebacterium* *glucuronolyticum,* as a common bacterial species found in semen of both healthy individuals and individuals with prostatitis [63, 64].

To determine the bacterial profile of semen in men with and without prostatitis, Illumina (HiSeq2000) sequencing was used. *Corynebacterium was* detected in 4.3% of healthy individuals, and in 6.6% of individuals with prostatitis, while *Corynebacterium seminale* was detected in 2.1% of healthy individuals and 4.6% of individuals with prostatitis [64]. Similarly, a series of studies done by [63, 65] determined *Corynebacterium* and *Corynebacterium seminale* to be prevalent in semen samples of men with and without prostatitis.

Stored semen was used to analyze the relationship between Human papillomavirus (HPV) and bacteria in semen. HPV-positive semen samples presented the following genera in significant abundance: *Streptococcus* (*p* = 0.0058), *Peptostreptococcus* (*p* = 0.012), and *Moraxellaceae* (*p* = 0.028). *Delfia*, *Streptococcus, Anaerococcus*, *Corynebacterium*, *Prevotella*, *Peptoniphilus*, *Dialister*, *Finegoldia*, *Bifidobacterium,* and *Propinoibacterium* were also detected among HPV-positive semen samples [72]. HIV-positive individuals displayed a lowered semen microbiome diversity and richness which improved after 6 months of ART administration [73].

Additional studies are required to confirm specific bacterial species for identification of semen.

#### Urine

The healthy urinary bacteriome, consist of common genera *Lactobacillus*, *Corynebacterium*, *Staphylococcus*, *Prevotella*, and *Streptococcus*. Although *Lactobacillus crispatus*, *Lactobacillus jensenii,* and *Atopobium vaginae* have been found in the urine of females, it could be due to the interaction between urine and the vaginal fluid.

Metagenome sequencing showed that the microbiome of urine contained a greater abundance of *Actinotignum*, *Aerococcus*, *Atopobium*, *Facklamia*, *Gardnerella*, *Lactobacillus*, *Megasphaera*, *Oligella*, Prevotella, and *Streptococcus* in healthy individuals [74].

In a first-ever study of characterization of urine microbiome of children by metagenomic sequencing, [75] observed differences between urine bacterial composition of healthy male and female children (*n* = 40) aged 1–18 years. Girls exhibited significantly higher levels of *Firmicutes*, whereas boys had significantly higher levels of Actinobacteria. The genus *Anaerococcus* dominated the urinary bacteriome of healthy girls, with a significant increase in *Anaerococcus prevotii*, *Anaerococcus vaginalis*, and *Veillonella parvula* (*p*-value < 0.001) when compared to that of boys. An increased relative abundance of *Xylanimonas* and *Arthrobacter*, with a significantly high abundance of *Arthrobacter* sp. FB24 (*p*-value 0.0028) and *Arthrobacter aurescences* (*p*-value 0.015), was observed in boys.

##### Disease Studies

*Escherichia coli* have been found in urine and is the most common cause of complicated urinary tract infections [59], while *Pseudomonas aeruginosa* has been found to cause uncomplicated urinary tract infections. *Pseudomonas aeruginosa* is responsible for 7–10% of urinary tract infections within hospitalized patients [61, 62].

RT-PCR was used to successfully detect *Escherichia coli* in the urine of patients suffering with urinary tract infections. *Escherichia coli* was found in 56% of the samples, with 100% sensitivity and 89.4% specificity (75). Individuals with infectious and inflammatory processes of the urinary tract presented the following genera in abundance *Acidovorax*, *Alloscardovia*, *Epilithonimonas*, *Lachnospira*, *Peptostreptococcus*, *Pseudomonas*, *Rhodanobacter*, *Riemerella*, *Sphingobium,* and *Ureaplasma* [74] (Table S1).

## Discussion and Conclusions

Rapid developments in next-generation sequencing technologies such as 16S amplicon sequencing and shot gun metagenomic sequencing, bioinformatics and microbial sampling have promoted the popularity of microbial profiling in recent years [76, 77]. Among various applications of microbial profiling, identification of forensically relevant body fluids is of interest in the present narrative review. Sometimes crime scenes render degraded DNA, and conventional methods of body fluid identification is not feasible. In such scenarios, microbial profiling for body fluid identification is an efficient approach, which could also link a suspect to a crime scene [35, 78]. Till date, microbiome studies have reported body fluid-specific bacteria at both genus and species levels [79]. The present review aimed to collate bacterial species which have been reported to be abundant in saliva, semen, vaginal fluid and urine across geographical locations in a healthy state, both in forensic and non-forensic studies. In addition, bacterial species identified in oral, vaginal, urinary, and male reproductive organ infections have also been mentioned. The most widely reported saliva-specific bacterial species are *Streptococcus salivarius*, *Prevotella melaninogenica*, and *Neisseria flavescens* found in both healthy and diseased individuals, with *Fusobacterium nucleatum* associated with an increased diseased state. *Lactobacillus crispatus* and *Lactobacillus iners* are frequently dominant in the vaginal microbiome of healthy women. *Atopobium vaginae, Prevotella bivia,* and *Gardnerella vaginalis* are more prevalent in women with bacterial vaginosis.

Bacterial genera in semen and urine have been reported, but species-level identification by 16S rRNA microbial profiling is still not well-defined. Though more genera are identified overall by 16S rRNA gene profiling [80], 16S rRNA sequencing tends to offer less resolution for detecting changes at the species level and cannot detect strain-level changes [81–83]. 16S rRNA gene-based amplicon sequencing is also prone to technical biases, such as the efficiency of the DNA extraction method and performance of the primer pair used for PCR amplification, which may prevent accurate prediction of bacterial taxonomic ranks present in a sample, especially when aiming at species-level resolution [84, 85].

However, 16S rDNA sequencing has been reported to be more sensitive than whole genome sequencing (WGS) for urine metagenome analysis because WGS uses limited technical amplification of the nucleic acid content in the sample, thus, more closely reflecting the proportionate biomass contributed by microbes in urine [74]. Additionally, metagenomic sequencing would not be beneficial with samples containing low abundance of microflora. In such cases, deep 16S RNA sequencing would be preferred.

The building of knowledge on bacteria specific to human body fluids serves as a major advantage for the future of forensics. However, incorporation of body fluid microbiome typing into forensic investigations still presents several challenges. As emphasized by [11], in the case of microbiome sequencing to identify body fluids, a statistical testing framework based on the likelihood ratios of competing hypotheses is expected to be of great value and, thus, requires further exploration. Concurrently, bacterial markers that provide strain-level resolution are preferred over 16S rRNA gene data because of the higher level of resolution achieved [11].

The identification of species-specific bacteria allows development of antimicrobial peptides which could be used as sensing elements for body fluid biosensor design [86]. Forensic biosensors demonstrate sensitive target identification and ease of detection is enhanced via the incorporation of nano materials. [87] designed an optical biosensor using a bovine serum albumin (BSA) stabilized Silicon Carbide (SiC) nanoparticles (SiC@BSA NPs) conjugated with antibacterial peptide GH12 to detect the oral bacteria *Streptococcus salivarius.*

The human microbiome is diverse and is greatly influenced by diet, ethnicity, disease status and lifestyle. The present review does mention differences in ethnicity and disease state; however, no other factors affecting the microbiome have been discussed or elaborated. Another limitation of the present review is that the bacterial species for diseased state were selected based on studies covering relatively small subsets of diseases. They may not be representative of a general diseased state, given that infections (and associated antibiotic treatments) could drastically shift the microbial composition equilibrium.

The selected species must, therefore, be tested in a large population study, taking into consideration several environmental and pathophysiological factors, before they are used in a forensic setting. Microbiomes also differ significantly in males and females [9, 75], hence this should also be factored in when undertaking microbial profiling studies.

Most of the studies documented on core microbiomes of body fluids targeted only one or two body fluids [17, 19, 88, 89]. Hence, future research should target all forensically relevant body fluids in a single study to ascertain the specificities of bacterial species. For forensic applications, methods should be modified to allow simultaneous microbial profiling and DNA typing in a single setting. Additionally, to avoid false positives, clusters of microbial species should be targeted instead of one or two single species.

The use of microbiome for identification of individuals could also be possible in the near future; however, it would require development and maintenance of specific microbial databases consisting of information on variables which affect microbial profiles such as ethnicity, diet, disease status, etc. Additionally, collection, storage and analysis methods of body fluids for microbial profiling need to be standardized due to the dynamic nature of microbiomes. Inclusion of microbial data into forensic investigation would also require robust statistical tests which hold value in a court of law.

Machine learning and classification methods as applied in microbial forensic research may be useful in identifying potential contamination sources and labeling errors in samples of forensic relevance [12, 15, 90]. It is also recommended that the applicability of microbial markers should be evaluated on mixed and aged samples [4]. Developmental validation of microbial profiling methods, starting from sample collection and storage to data analysis would make the procedure apt for forensic applications and complement DNA-based identification methods for body fluids. Development of population and region-specific microbial database will greatly aid in individualization of forensic samples. The emerging bioinformatics methods for analyzing metagenomics and 16S rRNA amplicon data will facilitate higher accuracy and resolution in defining the microbiome of each body fluid.

## Supplementary Information

Below is the link to the electronic supplementary material.

Supplementary file1 (DOCX 23 kb)

## Data Availability

Not applicable.
