# Supplementary material for: A Review on Microbial Species for Forensic Body Fluid Identification in Healthy and Diseased Humans
Source: Curr Microbiol. 2023 Jul 25;80(9):299. doi: 10.1007/s00284-023-03413-x (PMC10368579; doi:10.1007/s00284-023-03413-x)
Supplement: Supplementary file 1 — Supplementary file1 (DOCX 23 kb) [file 284_2023_3413_MOESM1_ESM.docx]

**A review on microbial species for forensic body fluid identification in healthy and diseased humans**

**Current Microbiology**

Mishka Dass *a, Yashna Singh *b, Meenu Ghai #c

Department of Genetics, School of Life Sciences, University of KwaZulu Natal – Westville

Campus, Private Bag X 54001, Durban, KwaZulu Natal, South Africa

a dassmishka@gmail.com

b yashnasingh1616@gmail.com

c ghai@ukzn.ac.za

*Co-first authorship

#Corresponding author

Corresponding author’s email address: ghai@ukzn.ac.za

Table S1: Bacterial genera and species associated with five forensically relevant body fluids, saliva, semen, vaginal fluid, menstrual blood and urine described in the text.

| **Body fluid** | **Bacterial genera and species** |
| --- | --- |
| **Saliva** | *Fusobacterium nucleatum* [40,43], *Lactobacillus fermentum* [40] , *Lactobacillus salivarius* [30,39] *Lactobacillus rhamnosus* [39], *Prevotella* *melaninogenica* [31,29,34], *Streptococcus mutans* [8,39], *Streptococcus salivarius* [3,8,11,12,15, 20,36], *Streptococcus sanguinis*[3], *Streptococcus mitis*[3]*,* *Streptococcus oralis* [8]*, Granulicatella* [25, 33,34]*, Rothia* [25, 33, 34,35]*, Selenomonas* [32]*, Streptococcus* [25, 32,34,35]*, Veillonella , Neisseria* [25, 32,35,41]*, Prevotella* [25,32,35,41]*, Fusobacterium* [25,31]*, Prevotella nigrescens* [31]*, Neisseria meningtidis* [31]*, Neisseria flavescens* , *Rothia mucilaginosa* [33]*, Porphyromonas* [34,35,41]*,Porphyromonas pasteri* [36]*, Parvimonas* [41]*, Granulicatella adiacens* [33]*, Neisseria subflava* [3] |
| **Vaginal Fluid** | *Lactobacillus crispatus* [12,15, 46, 49, 51,58,59] *Lactobacillus jensenii* [58]*, Lactobacillus iners* [46,52 ,58] *, Lactobacillus gasseri* [46,51,58]*, Lactobacillus coleohominis* [57] *, Lactobacillus vaginalis* [58]*, Prevotella bivia* [55,57]*, Prevotella amnii* [46]*, Atopobium vaginae* [46,49, 58,59] *, Gardnerella vaginalis* [45, 49, 58,59], *Lactobacillus, Gardnerella, Atopobium, Vibrio* [49] |
| **Menstrual Blood** | Lactobacillus gasseri [15] , Lactobacillus crispatus , Lactobacillus iners , Lactobacillus jensenii [9], Gardenella vaginalis [4, 63], Prevotella [63] |
| **Semen** | *Corynebacterium* [5, 64, 66*,*68], *Prevotella* [64, 68], *Staphylococcus* [5, 64] , *Streptococcus* [5*,*68], *Peptoniphilus* [68], *Propionibacterium, Anaerococcus* [68], *Corynebacterium seminale* [65,66], *Corynebacterium singular*, *Corynebacterium minutissimum, Dermbacter hominis* [5], *Finegoldia, Veillonella, Prevotella , Delifa* , *Bifidobacterium, Dialister* [68] |
| **Urine** | *Lactobacillus*, *Prevotella, Streptococcus* [70], *Anaerococcus Anaerococcus prevotii, Anerococcus vaginalis, Xylanimonas, Arthrobacter, Arthrobacter aurescences* [70, 71], *Escherichia coli* [75], *Pseudomonas aeruginosa* [73], *Gardnerella, Actinotigum, Atopobium, Facklamia, Megasphaera oligella ,* *Acidovorax, Alloscardovia , Epilithonimonas, Lachnospira, Peptostreptococcus , Pseudomonas, Rhodanobacter, Riemerella, Sphingobium, Ureaplasma* [70] |
